# Leveraging patient data to detect systematic shifts in daptomycin susceptibility testing associated with reduced prescribing

**DOI:** 10.1128/jcm.01510-25

**Published:** 2026-02-04

**Authors:** Mark A. Zaydman, Laurel Glaser, Daniel S. Herman, Mason Kressloff, Vahid Azimi, Christine R. Lockowitz, Rebekah E. Dumm

**Affiliations:** 1 Department of Pathology & Immunology, Washington University School of Medicine 12275 , St. Louis, Missouri, USA; 2 Department of Pathology and Laboratory Medicine, Perelman School of Medicine, University of Pennsylvania 14640 , Philadelphia, Pennsylvania, USA; 3 Department of Laboratory Medicine and Pathology, Alameda Health System 440324, Oakland, California, USA; 4 Department of Pharmacy, St. Louis Children’s Hospital 819401, St. Louis, Missouri, USA; Maine Medical Center Department of Medicine, Portland, Maine, USA

**Keywords:** antibiogram, QC, daptomycin, antimicrobial susceptibility testing

## Abstract

Systemic shifts in antimicrobial resistance rates can be due to epidemiologic shifts in microbial susceptibility patterns or artifactual shifts introduced by technical biases in antimicrobial susceptibility testing (AST)—both ultimately leading to changes in antimicrobial prescribing. To reduce technical variability, quality control (QC) criteria for AST are published by manufacturers and standards organizations. However, traditional QC metrics, in isolation, are fallible. In this study, we describe a systematic shift in daptomycin AST results between 2022 and 2025 in isolates tested in two independent health systems. Comprehensive analysis of clinical isolate AST results and retrospective mining of QC data from this period revealed a subtle shift that led to a 5%–22% decrease in overall susceptibility rates for certain organisms, most notably *Enterococcus faecium*. As daptomycin is a key treatment option for these difficult-to-treat infections, this increase in resistance rates paralleled a decrease in prescribing daptomycin for infections with these organisms. Importantly, this trend was undetectable through routine QC processes and only became apparent through systematic review of patient data. Our findings highlight the opportunity to integrate routine patient data analysis into microbiology QC practices to enhance detection of subtle but clinically relevant changes in AST performance.

**IMPORTANCE:**

In this study, we report a critical incident of technical variability using daptomycin gradient diffusion methodology that was undetectable using routine quality control metrics. More broadly, this study underscores the opportunity to incorporate additional modalities, such as clinical patient results, into a comprehensive quality assurance plan to ensure high-quality antimicrobial susceptibility testing results. Given the dynamic spread of multidrug resistance in bacteria, accurate susceptibility testing results are critical to identify and respond to shifts in local epidemiology.

## INTRODUCTION

Ensuring the accuracy and reliability of antimicrobial susceptibility testing (AST) is crucial for effective targeted treatment of infections. Quality control (QC) and quality assurance (QA) metrics in AST involve reagent testing for new lots and shipments, organism comparison to predictable resistance profiles, and the regular review of cumulative antibiogram data to monitor trends over time. These practices are designed to detect analytical deviations and maintain the integrity of test results.

Quality control of AST for new lots of reagents is performed by both the manufacturer and end user laboratories. While manufacturers perform extensive QC for reagents before releasing product to the market, end user QC focuses on reagent quality after shipping and storage (1). End user QC ranges are set by standards organizations such as CLSI or EUCAST for well-characterized microbial strains, to detect anomalies in protocols and reagents across all laboratories rather than dynamic QC ranges per reagent lot employed in other lab disciplines (2, 3). These ranges are agnostic of categorical interpretation and institution-specific variation and, thus, serve as only one aspect of a robust QC program.

Cumulative analysis of antimicrobial susceptibility data in the form of an antibiogram allows monitoring of broad-scale, population-level emergence of resistance over time and can also serve as a QA metric to identify shifts. While antibiograms are typically produced on an annual basis (4), they can provide unique insights when employed more frequently, accessibly, or on a more granular unit level (5, 6). Antibiograms are especially valuable for organism-antibiotic combinations for which there are known technical challenges or first-line antibiotics that are recommended in local empiric therapy guidelines.

Daptomycin, a cyclic lipopeptide, has activity against many significant Gram-positive pathogens including *Staphylococcus* and *Enterococcus* spp. that are susceptible or resistant to vancomycin (7). Since its introduction in 2003 and the advent of generic formulations in 2015, daptomycin has become a first-line therapy for resistant Gram-positive infections (8). Available antimicrobial susceptibility testing (AST) methods for daptomycin include gradient diffusion and automated platforms (9, 10) and are routinely performed in many clinical laboratories. Despite this widespread use, daptomycin susceptibility testing has proven technically challenging, with false nonsusceptible results by several methods (11, 12), and more reliable results noted with certain manufacturers (13). Other studies have noted imprecision is isolates containing mutations in the *liaFSR* system, which make establishment of revised breakpoints challenging for these organisms (14). Despite these challenges and in the context of widespread use, resistance rates of most Gram-positive organisms to daptomycin have remained under 10% (15).

In this study, two academic hospitals similarly reported historical gram-positive daptomycin susceptibilities reliably at rates >90%. However, in compiling 2023–2024 antibiograms, a drop was observed in the reported daptomycin susceptibility which was reflected in decreased daptomycin prescribing. This prompted a thorough investigation, which ultimately identified a single doubling dilution shift caused by an analytical, rather than epidemiologic, mechanism. Despite performing QC according to current guidelines, this issue was not detected in real-time, highlighting important limitations of current standard-of-practice QC methods. Our findings suggest that incorporating a patient data would contribute to a comprehensive quality assurance program for antimicrobial susceptibility testing.

## MATERIALS AND METHODS

### Generation of annual antibiograms

Antibiograms were generated using clinical isolates collected and tested from Barnes-Jewish Hospital (BJH) and St. Louis Children’s Hospital (SLCH) using in-house software. Antibiograms from the Hospital of the University of Pennsylvania (HUP) clinical microbiology laboratories were generated using ILÚM Insight from Infectious Disease Connect, Inc. Only the first isolate per patient, based on collection time, was included in the generation of the antibiograms. BJH performs routine daptomycin testing for all *Enterococcus* isolates and *Staphylococcus* isolates from sterile sources. HUP performs daptomycin testing on VRE and MRSA from blood and sterile body fluid and by request for resistant gram-positive organisms from other sources.

### Daptomycin antimicrobial susceptibility testing

Daptomycin was tested against gram-positive bacteria, including *Staphylococcus epidermidis, Staphylococcus aureus, Enterococcus faecalis,* and *Enterococcus faecium,* using bioMerieux ETESTs (catalog 412323/423813) following manufacturer instructions. Both laboratories utilize up-to-date CLSI M100 interpretive criteria for daptomycin, most recently the 35th edition (3), but breakpoints were initially published in M100 17th edition for *Staphylococci* and M100 30th edition for *Enterococcus* spp, and *E. faecium*. Notably, the HUP clinical laboratory also confirmed a subset of daptomycin-resistant *Staphylococci* and *Enterococci* using the Sensititre panel GPALL3F. Therefore, their summary antibiogram includes data from both methods.

To compare lot-to-lot reproducibility, daptomycin was retested on a subset of frozen clinical isolates using contemporary lots of gradient diffusion strips. This cohort included 25 resistant isolates of *S. epidermidis, S. aureus, E. faecalis,* and *E. faecium* originally tested during standard of care in the BJH clinical microbiology laboratory between June 2023 and January 2024. Isolates were thawed, subcultured, and tested on BD BBL Mueller Hinton agar with bioMerieux ETESTs (catalog 412323/423813, lot 1010462780). As a control, repeat vancomycin testing was performed on a subset of isolates for which prior vancomycin MIC data were available using bioMerieux ETESTs (catalog 412486/423788). No MIC data were available for analysis of *Enterococcus* spp. since testing of 2023 isolates was performed using disk diffusion.

### Time series segmentation based on clinical isolate susceptibilities

Daptomycin susceptibility results between 2022-01-01 and 2024-12-31 at BJH and SLCH were extracted from the laboratory information system (Cerner Millenium, Oracle, Austin, TX). The first isolate per organism and patient encounter was defined as the index isolate and aggregated for the BJH data. Change point analysis was implemented using the *ruptures* library v1.1.9 in Python3 using the dynamic programming implementation with the L1 function model, a value of 2 for the number of breakpoints, and the default parameters values for the min_size (min_size=2) and jump (jump=5) parameters. Inputs to the algorithm consisted of the rolling 30-day susceptibility rates centered on each day in the study period for clinical isolates of *S. epidermidis, S. aureus, E. faecalis,* and *E. faecium*. All four time series data sets were considered simultaneously in model fitting. The breakpoints identified were used to segment the study period into three epochs. For each organism and epoch, the rate at which clinical isolates were reported as “Susceptible” or “Susceptible Dose Dependent” and the median MIC values were estimated and compared with the initial epoch as the reference.

### Analysis of trends in QC data

Laboratory QC data were retrieved from the laboratory information system for BJH and from paper records at HUP. Both laboratories performed QC using *Staphylococcus aureus* ATCC 29213 (acceptable range 0.12–1 µg/mL) (3). The BJH clinical microbiology laboratory performed QC testing daily on primarily BD BBL Mueller Hinton media (catalog 221177). The HUP clinical microbiology laboratory performed weekly QC after implementing reduced frequency antimicrobial susceptibility testing on REMEL Mueller Hinton agar (catalog R04050). The median QC values and failure rates were aggregated, rounded to the nearest doubling dilution and compared across the identified epochs.

### Local daptomycin prescribing practices at BJH & SLCH

BJH and SLCH both have antimicrobial stewardship programs (ASPs), which monitor daptomycin utilization. At BJH, providers can order daptomycin without prior approval, but all orders are evaluated by the ASP team within 72 h for appropriateness, with recommendations to continue or change therapy performed at that time. At SLCH, daptomycin always requires prior approval by the ASP team or on-call Infectious Diseases (ID) provider. At both BJH and SLCH, the ID consult teams can initiate daptomycin without ASP prior authorization. Additionally, BJH and SLCH perform prospective audit of antimicrobial prescribing with feedback to primary teams Monday through Friday.

### Analysis of trends in daptomycin usage at BJH & SLCH

All medication administrations for the patient encounters represented in the AST data set with a therapeutic class of “antibiotic” were extracted from the electronic health record reporting databases at BJH (EPIC, Verona, WI). Actions were filtered to those indicating that the medication was not just prescribed but administered to the patient, including “given,” “new bag,” “bolus,” “push,” “given by other,” “new syringe/cartridge,” “given during downtime,” “started during downtime,” “medication applied,” “bolus from bag,” “anesthesia bolus,” “self-administered via pump,” and “pump refill.” The number of antibiotic days was defined as the number of unique calendar dates during which the patient received an administration of the medication. The median number of antibiotic days per patient encounter was aggregated for each drug-bug-epoch combination. Vancomycin not susceptible (VNS) was defined by a clinical result interpretation of “intermediate” or “resistant.” Methicillin resistance was defined by a cefoxitin result interpretation of “resistant.”

### Statistics

Rates are presented with a 95% confidence interval defined using the proport_confint method of the statsmodel library (Python3) and compared using a chi-squared test implemented using the chi2_contingency method of the statsmodel library. Median values are presented with an interquartile (IQR) representing the 25th–75th percentile range and were compared using a Mann-Whitney *U* test implemented using the mannwhitneyu method of the scipy.stats package. A Bonferroni correction was used to control the family-wise error rate when multiple (*m* = 2) comparisons were made to a baseline period, resulting in a corrected significance threshold of *α* = 0.05/2 = 0.025.

## RESULTS

**Fig 1:**
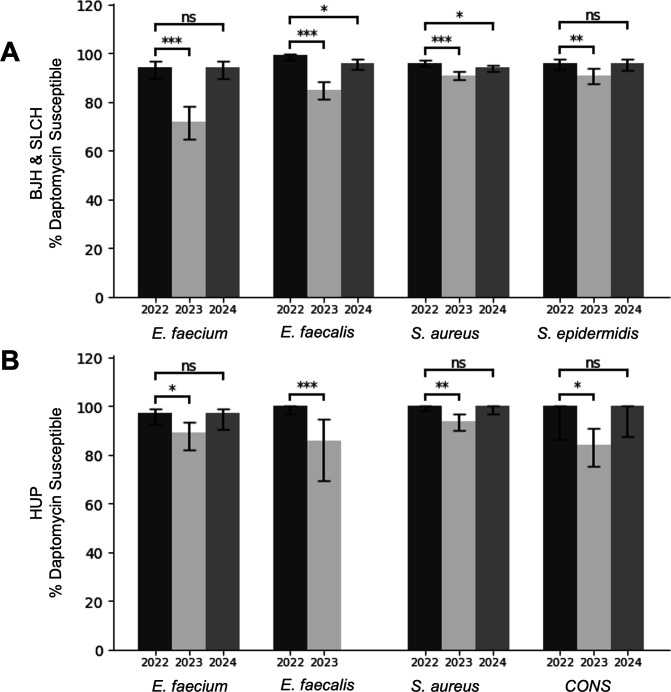
Daptomycin susceptibility rates for gram-positive organisms reported in the 2022, 2023, and 2024 antibiograms for (**A**) Barnes Jewish Hospital and St. Louis Children’s Hospital (BJH & SLCH) or (**B**) The Hospital of the University of Pennsylvania (HUP). “ns” = not significant, * = *P* < 0.05, ** = *P* < 0.01, *** = *P* < 0.001 by chi-squared test. Insufficient data were available to graph HUP *E. faecalis* isolates from 2024.

In the published 2022 annual antibiograms, >90% daptomycin susceptibility was observed among gram-positive clinical isolates at BJH & SLCH. Over the following years, however, reported daptomycin susceptibilities decreased in 2023 and rebounded in 2024 (Fig. 1A; Table S1). This trend was most pronounced for *E. faecium* for which the susceptibility rate decreased from 94% in 2022 to 72% in 2023 (*P* < 0.0001), and then increased back to 94% in 2024, which was no longer significantly different compared to 2022 (*P* = 0.9). A similar pattern was observed for *E. faecalis*, *S. aureus*, and *S. epidermidis*. Remarkably, the HUP antibiograms demonstrated a temporally associated decrease and subsequent rebound for daptomycin susceptibility to *E. faecium*, *E. faecalis*, *S. aureus*, and coagulase negative *Staphylococci* (CONS) (Fig. 1B; Table S1).

**Fig 2:**
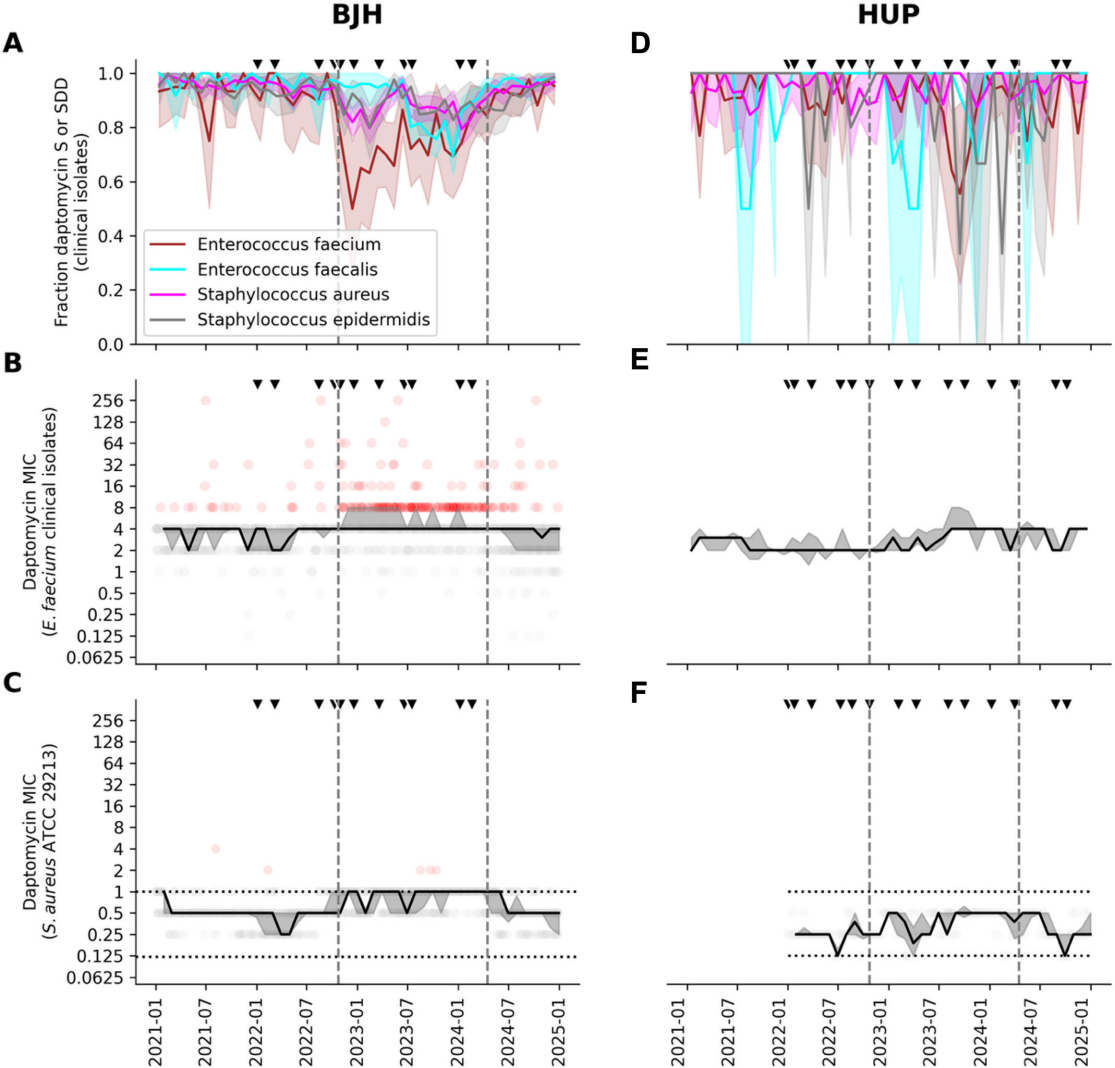
Daptomycin AST results at BJH and HUP. (**A, D**) The monthly fraction of index clinical isolates (e.g., first per patient encounter) of *Enterococcus faecium, Enterococcus faecalis, Staphylococcus aureus,* and *Staphylococcus epidermidis* and testing susceptible (S) or susceptible dose-dependent (SDD). (**B, E**) Daptomycin MIC values for index *Enterococcus faecium* clinical isolates. (**C, F **) Daptomycin MIC values for the *Staphylococcus aureus* ATCC 29213 quality control strain. In panel A, the shaded regions indicate the 95% confidence interval. In panels **B** and **C**, marker color indicates a result categorized as S or SDD (gray) or not susceptible (red), and lines and shaded regions indicate the monthly median and interquartile range (25th–75th percentile), respectively. In all panels, the vertical dashed lines represent change points determined for the data in the left side of panel **A**, and the triangle markers denote the timing of institution-specific daptomycin ETEST lot changes. Horizontal lines in **C** indicate the acceptable CLSI QC range.

The fraction of daptomycin susceptible index clinical isolates from BJH suggested a shift toward lower monthly susceptibility rates toward the end of 2022, with a reversion toward baseline in early 2024 (Fig. 2A). To objectively annotate these shifts, a change point analysis model was simultaneously fit to the 30-day moving average susceptibility rates calculated separately for index isolates of *E. faecium, E. faecalis, S. aureus,* and *S. epidermidis*. This identified the dates of 2022-10-24 and 2024-04-16 as change points, thereby defining three distinct time epochs denoted with dashed lines in Fig. 2: (i) the “baseline” epoch (2021-01-01 to 2022-10-24), (ii) the “affected” epoch (2022-10-24 to 2024-04-16), and (iii) the “recovery” epoch (2024-04-16 to 2024-12-31).

In the baseline epoch, 93.6% of BJH *E. faecium* clinical isolates tested susceptible to daptomycin (Fig. 2A; Table S2). This decreased significantly to 74.6% (*P* < 0.0001) in the affected epoch and then reverted back to 93.4% in the recovery epoch (*P* = 1.000 vs baseline). Similar trends were observed for *E. faecalis*, *S. aureus*, and *S. epidermidis*. Likewise, for BJH index *E. faecium* clinical isolates in the baseline epoch, the median minimum inhibitory concentration (MIC) of daptomycin was 4 µg/mL with an interquartile range (IQR) of 2–4 µg/mL (Fig. 2B; Table S2). While the median remained consistent, there was a subtle, but statistically significant increase in IQR to 4–8 µg/mL (*P* < 0.0001) in the affected epoch, which reverted to 2–4 µg/mL (*P* = 0.952 vs baseline). Similar shifts in both median and IQR MIC values were observed for *E. faecalis*, *S. aureus*, and *S. epidermidis* clinical isolates. 

This one doubling dilution shift was mirrored in the QC results at BJH (Fig. 2C; Table S1) where the median daptomycin MIC for the *S. aureus* ATCC 29213 QC strain went from 0.5 µg/mL in the baseline epoch to 1.0 (*P* < 0.0001) and then back to 0.5 (*P* = 0.132) in the affected and recovery epochs, respectively. There were five, five, and zero QC failures in the baseline, affected, and recovery epochs, respectively, which were all resolved by repeat testing alone.

Change points defined by the BJH clinical susceptibility rates were used to investigate correlations in HUP clinical and QC data (Fig. 2D through F; Table S1). Daptomycin susceptibility rates at HUP exhibited a similar trend where susceptibility rates decreased in the affected epoch before rebounding in the recovery epoch; however, these changes did not reach statistical significance. For example, the susceptibility rate of *E. faecium* dropped from 94% at baseline to 90% (*P* = 0.1 vs baseline) in the affected epoch and then increased back to 92% (*P* = 0.7). These changes in reported susceptibilities were driven by an increase in the median MIC values during the affected epoch, as seen for *E. faecium* (baseline: 2 µg/mL, affected: 4 µg/mL [(*P* < 0.001], recovery: 4 µg/mL [*P* < 0.001]), and similarly for *E. faecalis*, *S. aureus*, and CONS. There was one HUP QC failure during the study period, which occurred during the affected epoch.

**Fig 3:**
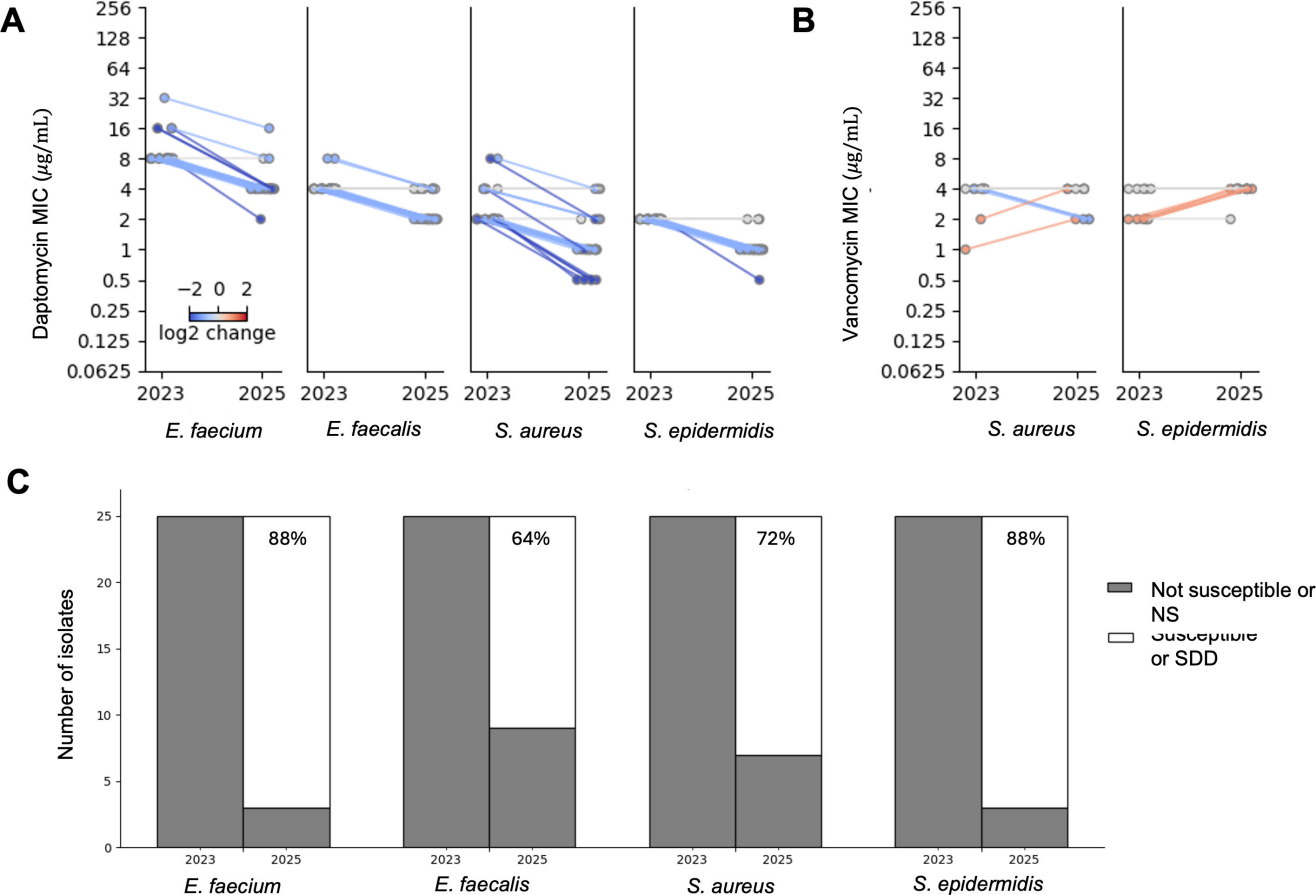
Repeat AST results for clinical isolates tested during the affected epoch. (**A**) Daptomycin MIC values of select not susceptible clinical isolates as reported during the affected epoch (2023) and then observed upon repeat gradient diffusion testing using a new reagent lot (2025). Colors indicate the doubling dilution change. (**B**) Vancomycin MIC values of clinical isolates as initially reported and then retested for a subset of isolates in panel A. (**C**) Interpretation of the daptomycin susceptibilities for the clinical isolates in panels A*—E. faecium* shifted from resistant to susceptible dose-dependent. *E. faecalis* and *Staphylococci* shifted from not susceptible (intermediate/resistant/nonsusceptible) to susceptible.

A subset of 100 clinical isolates of *E. faecium*, *E. faecalis*, *S. aureu*s, and *S. epidermidis* that tested as not susceptible at BJH during the “affected” epoch in 2023 were retested using a new reagent lot of ETEST gradient diffusion strips available in 2025 (Fig. 3A). The median change in MIC was –1 doubling dilutions for all organisms (*P* < 0.001 by Wilcoxon test). As a control, vancomycin susceptibility for any of this cohort with prior vancomycin MIC results was retested using a 2025 reagent lot of ETEST gradient diffusion strips. (Fig. 3B). For these, the median change in MIC was not significantly different from zero for *S. aureus* (*n* = 10, *P* = 1) and for *S. epidermidis* (*n* = 11, *P* = 0.0625). After applying interpretive criteria, 22/25 (88%) of *E. faecium* initially reported as resistant were interpreted as susceptible dose-dependent. Similarly, 16/25 (64%) of *E. faecalis* shifted from intermediate to susceptible. Lastly, 18/25 (72%) of *S. aureus*, and 22/25 (88%) of *S. epidermidis*, shifted from intermediate or resistant to susceptible upon retesting (Fig. 3C).

Daptomycin administration record data were collected for all encounters with a clinical isolate of VNS *E. faecalis*, and VNS *E. faecium*, MRSA, and MRSE identified on culture (Table 1; Fig. S1). These results are guideline-directed use cases for daptomycin at BJH and SLCH. The median [IQR] number of daptomycin days per patient encounter with a VNS *E. faecium* clinical isolate decreased significantly from 4 [0–10] days in the baseline epoch to 0 days [0–2] (*P* = 0.012) in the affected epoch and remained significantly depressed at 0 [0–0] days (*P* < 0.0001) in the recovery epoch. A similar trend was observed for VNS *E. faecalis*, for which the median number of daptomycin days decreased from 0 [0–4] to 0 [0–0] (*P* = 0.012) and remained as 0 [0–0] (*P* = 0.009). The median [IQR] number of daptomycin days was 0 [0–0] at baseline for both MRSA and MRSE and did not decrease significantly in the affected epoch.

**TABLE 1:** Daptomycin susceptibility and prescribing rates for resistant gram positive isolates

	2021-01-01 to 2022-10-24			2022-10-24 to 2024-04-16			2024-04-16 to 2024-12-31		
	“Baseline Epoch”			“Affected Epoch”			“Recovery Epoch”		
	Value	*n*	*P*	Value	*n*	*P*	Value	*n*	*P*
VNS *E. faecium* % susceptible [CI]	92.5 [89.1–95.8]%	239	1	71.9 [67.4–76.4]%	381	<0.001	95.1 [92.4–97.8]%	244	0.3
VNS *E. faecalis* % susceptible [CI]	94.4 [88.3–100.0]%	54	1	95.3 [90.9–99.8]%	86	1	94.9 [87.9–100.0]%	39	1
MRSA % susceptible [CI]	95.0 [93.9–96.0]%	1,546	1	84.7 [82.7–86.7]%	1,273	<0.001	93.3 [91.3–95.3]%	583	0.2
MRSE % susceptible [CI]	95.5 [94.3–96.7]%	1,164	1	88.0 [85.9–90.1]%	932	<0.001	92.0 [89.3–94.6]%	398	0.009
VNS *E. faecium* median MIC [IQR]	4 [2–4]	306	1	4 [4–8]	442	<0.001	4 [2–4]	271	0.3
VNS *E. faecalis* median MIC [IQR]	1 [1–2]	62	1	2 [1–2]	95	<0.001	1 [1–2]	42	0.8
MRSA median MIC [IQR]	0.5 [0.5–1]	2,061	1	1 [1–1]	1,716	<0.001	0.5 [0.5–1]	759	0.2
MRSE median MIC [IQR]	0.5 [0.5–1]	2,169	1	1 [1–1]	1,729	1	0.5 [0.5–1]	691	1
VNS *E. faecium* median number days^*a*^ [IQR]	4 [0–10]	240	1	0 [0–2]	381	0.0118	0 [0–0]	243	<0.001
VNS *E. faecalis* median number days^*a*^ [IQR]	0 [0–4]	54	1	0 [0–0]	86	0.0118	0 [0–0]	39	0.009
MRSA median number days^*a*^ [IQR]	0 [0–0]	1,545	1	0 [0–0]	1,274	0.311	0 [0–0]	581	0.998
MRSE median number days^*a*^ [IQR]	0 [0–0]	1,166	1	0 [0–0]	933	0.76	0 [0–0]	395	0.007

^*a*^ Number of Daptomycin prescribing days.

## DISCUSSION

In this multi-center study, a transient decline in daptomycin susceptibility among gram-positive pathogens—*E*. *faecium*, *E. faecalis*, *S. aureus*, and *S. epidermidis*—isolated from cultures at Barnes-Jewish Hospital (BJH)/St. Louis Children’s Hospital (SLCH) and the Hospital of the University of Pennsylvania (HUP) was revealed across the annual antibiograms published for 2022, 2023, and 2024. Similar shifts from two geographically distinct institutions argued against a single-site phenomenon and several lines of evidence suggest an analytical artifact rather than a true epidemiological emergence of daptomycin resistance. First, a simultaneous rise in daptomycin resistance across multiple gram-positive organisms is unlikely, given that mechanisms of resistance are multifactorial and not readily exchanged between species (16). Additionally, a similar shift was observed for QC testing of similar magnitude and timing as the MIC shift for clinical isolates. Lastly, retesting a selection of clinical isolates from the affected epoch demonstrated lower daptomycin MIC values on contemporary lots of the same reagent.

A thorough investigation was conducted of the many potential sources of analytical variation in AST testing, including the reagents, diluents, media, workflows and procedures, incubators, and personnel (17). It was determined that variation in the daptomycin gradient diffusion strips was the most plausible source given the temporal dynamics and as the only shared reagent used at both BJH and HUP. However, a direct head-to-head comparison was impossible given the timing of the discovery and unavailability of previously used reagents. There was also no overlap in the reagent lot numbers used at BJH and HUP during the study period, and we did not have access to track lot numbers to shared manufacturing runs. We communicated our findings back to the test strip manufacturer and were informed of instrumentation changes during the months of the affected epoch that could have contributed to the observed shift.

While the magnitude of the daptomycin MIC shift was similar between BJH and HUP data sets, HUP susceptibility rates were less affected. Several technical factors might explain these differences, including institutional AST reading practices, differences in Mueller Hinton media manufacturers, and differences in isolate cohorts tested. While BJH tests all *Enterococci* and reports selectively, HUP selectively tests more resistant isolates which can skew antibiogram data. Lastly, HUP retested a subset of daptomycin nonsusceptible isolates using a secondary method by director review, which are included in aggregate antibiogram data (Fig. 1) and may temper the observed shift attributed to the gradient diffusion strips. Overall, BJH isolates test closer to the interpretive breakpoint at baseline explaining why a similar magnitude shift led to a greater fraction of BJH clinical isolates being categorized as not susceptible.

The affected epoch was associated with reduced daptomycin utilization for VNS *E. faecalis* and VNS *E. faecium* infections at BJH, indicating that, even though the absolute magnitudes of these changes were small, they had significant implications for patient management. The observed shift in prescribing behavior was likely due to both empiric and targeted therapy decisions and influenced by antibiogram distribution highlighting this concerning trend. The uncertainty in daptomycin susceptibility trends also likely influenced depressed prescribing practices throughout 2024 despite a return to higher susceptibility rates (18). This underscores the importance of these reports to inform institutional guidelines and supports the case for more real-time release of aggregate susceptibility results to identify trends.

**Fig 4:**
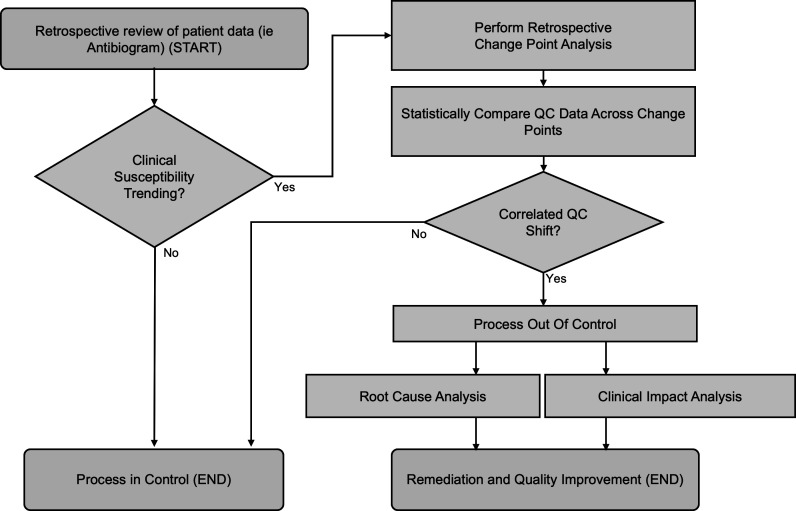
Framework for novel clinical AST quality management process beginning with periodic review of retrospective patient data.

The analyses described in this study suggest a novel clinical AST quality management process (Fig. 4), beginning with periodic review of retrospective patient data either via quantitative comparison of annual antibiograms or visual inspective of more granular time series data. A change in the clinically reported susceptibility rates triggers further investigation using retrospective change point analysis for quantitative correlation with QC trends. Identification of a temporally and directionally correlated QC shift suggests loss of process control and the need for root cause and clinical impact analyses. In contrast, the lack of a significant trend in the clinical data suggests, or the absence of a corresponding QC shift suggests no meaningful loss of process control (end-of-process). This method can retrospectively identify clinically meaningful analytical issues but are not ideal for prospective quality monitoring and, therefore, will not improve the timeliness of real-time error detection.

Several strategies to improve AST QC programs may be the subject of future investigation. First, a more frequent or rolling antibiogram might help to more quickly detect a potential issue (19). In this study, comparison of annual antibiograms was the first signal; however, the time lag to detection was unacceptably long. Currently, regulatory bodies mandate only an annual cadence (4), recognizing the challenges of aggregating robust statistics over shorter intervals, especially for rare bug-drug combinations or in a low-volume clinical context. Potential solutions might involve a rolling aggregate over the most recent 30 index isolates or a more robust statistical analysis that accounts for sample size and uncertainty in the rate estimate. Second, analyses might be expanded or normalized to increase sensitivity, considering susceptibility rates for all commonly clinically isolated species characterized using a specific drug, rather than each bug-drug combination independently. In the present study, a correlated decrease in daptomycin susceptibility across multiple gram-positive microorganisms was an essential clue to an analytical issue. Third, tracking MIC values, rather than a categorical SIR (Susceptible, Intermediate, Resistant) for clinical susceptibilities or a boolean In/Out for QC results might provide additional sensitivity. Fourth, clinical and QC strain data streams could be analyzed together in a multimodal approach to improve sensitivity and disambiguate analytical from epidemiological shifts. This could be implemented by visualizing both data streams on a consistent set of axes, as in Fig. 2, or by implementing a more sophisticated algorithmic approach such as real-time change point analysis. Furthermore, broadening this multimodal approach to include prescribing data, as in this analysis, could serve as an additional signal of a shift. Finally, vendors and laboratories could improve communication and collaboration to facilitate the identification of manufacturing defects and investigating how lot-to-lot variation does or does not impact clinical reporting and downstream medical decision-making.

An inherent challenge for employing these proposed methods is balancing sensitivity with specificity for shifts that are not explained by imprecision or random variation. Defining an appropriate threshold depends not only on the choice of analytical method but also on the settings of its hyperparameters. For example, using moving averages to flag process shifts in high-volume clinical chemistry testing requires selecting values for the truncation limits, block size, and control limits hyperparameters (20). The optimal values for these parameters may depend on laboratory-specific testing volumes, operational workflows, and clinical priorities. This variability makes it difficult to generalize these tools with additional local tuning that can further burden laboratories.

Several limitations of the current study should be acknowledged. First, we are not able to map the change points precisely to when a specific lot was implemented, and we did not have knowledge of which lots arose from the same reagent manufacturing run. Second, the change point analysis implementation required ad-hoc decisions for the number of breakpoints and the hyperparameters *jump* and *min_size*. Practically, this specific change point analysis approach is limited as it will not readily translate into a prospective monitoring tool. Finally, without access to broader data sets, we are unable to determine the true magnitude of this analytical issue. Similar trends spanning geographically distinct health systems suggest that the impact may have been widespread.

In summary, we describe an analytical issue in clinical daptomycin susceptibility testing that led to overreporting of daptomycin resistance and decreased prescription of daptomycin. These results demonstrate the current limitations of QC testing for AST methods and suggest several opportunities for improvement that warrant further investigation.

## SUPPLEMENTAL MATERIAL

Supplemental tables and figure jcm.01510-25-s0001.pdf Tables S1 and S2, and Figure S1.

ASM does not own the copyrights to Supplemental Material that may be linked to, or accessed through, an article. The authors have granted ASM a non-exclusive, world-wide license to publish the Supplemental Material files. Please contact the corresponding author directly for reuse.
