# Supplementary material for: Leveraging patient data to detect systematic shifts in daptomycin susceptibility testing associated with reduced prescribing
Source: J Clin Microbiol. 2026 Feb 4;64(3):e01510-25. doi: 10.1128/jcm.01510-25 (PMC12977467; doi:10.1128/jcm.01510-25)
Supplement: Supplemental tables and figure — Tables S1 and S2, and Figure S1. [file jcm.01510-25-s0001.pdf]

Supplemental Table 1: Summary of 2022-2024 Daptomycin Antibiograms for BJH & SLCH and HUP

		2022			2023			2024		
	**Organism**	**% sus[CI]**	**n**	**p**	**% sus[CI]**	**n**	**p**	**% sus[CI]**	**n**	**p**
**BJH & SLCH**	***E. faecium***	94[89.7-96.8]%	174	1	72[64.8-78.1]%	171	<0.001	94[89.6-96.5]%	196	0.9
	E. faecalis	99[97.2-99.7]%	314	1	85[81.1-88.1]%	404	<0.001	96[93.5-97.5]%	392	0.01
	S. aureus	96[94.7-97.1]%	1060	1	91[89.1-92.6]%	1029	<0.001	94[92.4-95.2]%	1059	0.03
	S. epidermidis	96[93.5-97.7]%	358	1	91[87.6-93.6]%	356	0.006	96[93.0-97.6]%	293	0.9
**HUP**	***E. faecium***	97[92.5-98.8]%	132	1	89[82.2-93.3]%	126	0.01	97[90.6-98.8]%	89	0.9
	E. faecalis	100[96.7-100.0]%	112	1	86[69.4-94.5]%	29	<0.001	NA	NA	NA
	S. aureus	100[97.9-100.0]%	181	1	94[89.9-96.7]%	190	0.001	100[96.8-100.0]%	115	1
	**CONS**	100[86.2-100.0]%	24	1	84[75.3-90.7]%	84	0.04	100[87.5-100.0]%	27	1

%sus = % susceptible isolates
[CI] = confidence intervals
CONS = Coagulase negative Staphylococci
NA = Insufficient data were available to graph HUP E. faecalis isolates from 2024.

Supplemental Table 2: Summary of 2022-2024 Daptomycin Susceptibility Rates & QC metrics for BJH & SLCH and HUP

	2021-01-01 to 2022-10-24 "Baseline Epoch"			2022-10-24 to 2024-04-16 "Affected Epoch"			2024-04-16 to 2024-12-31 "Recovery Epoch"		
	**value**	**n**	**p**	**value**	**n**	**p**	**value**	**n**	**p**
**Enterococcus faecium fraction susceptible %[CI]**	93.6[91.1-96.2]%	362	1	74.6[71.1-78.0]%	614	<0.001	93.4[90.9-96.0]%	366	1
**Enterococcus faecalis fraction susceptible %[CI]**	97.0[95.8-98.2]%	774	1	86.4[85.0-87.8]%	2312	<0.001	96.9[96.0-97.8]%	1485	1
**Staphylococcus aureus fraction susceptible %[CI]**	95.8[95.2-96.4]%	3864	1	87.0[85.8-88.2]%	3136	<0.001	94.9[93.8-96.1]%	1441	0.2
**Staphylococcus epidermidis fraction susceptible %[CI]**	95.0[93.9-96.0]%	1612	1	88.6[86.8-90.3]%	1296	<0.001	92.1[89.9-94.3]%	568	0.01
**Enterococcus faecium daptomycin MIC median[IQR]**	4[2-4]	461	1	4[4-8]	714	<0.001	4[2-4]	405	1
**Enterococcus faecalis daptomycin MIC median[IQR]**	1[1-2]	902	1	2[2-2]	2490	<0.001	2[1-2]	1579	<0.001
**Staphylococcus aureus daptomycin MIC median[IQR]**	0.5[0.5-1]	4990	1	1[1-1]	4075	<0.001	0.5[0.5-1]	1803	<0.001
**Staphylococcus epidermidis daptomycin MIC median[IQR]**	0.5[0.5-1]	3062	1	1[1-1]	2497	1	0.5[0.5-1]	998	1
**S. aureus ATCC 29213 daptomycin MIC median[IQR]**	0.5[0.5-0.5]	670	1	1[0.5-1]	554	<0.001	0.5[0.5-0.5]	260	0.1
**QC failure rate[CI]**	0.01[0.00-0.01]	5	1	0.01[0.00-0.02]	5	0.8	0.00[0.00-0.00]	0	0.2
**Enterococcus faecium fraction susceptible %[CI]**	94[91-97]%	230	1	90[86-94]%	215	0.1	92[87-98]%	91	0.7
**Enterococcus faecalis fraction susceptible %[CI]**	90[79-100]%	30	1	86[77-96]%	51	0.9	96[89-100]%	26	0.7
**Staphylococcus aureus fraction susceptible %[CI]**	95[92-97]%	395	1	95[92-97]%	409	1	96[0.94-0.99]	251	0.4
**Staphylococcus epidermidis fraction susceptible %[CI]**	93[85-100]%	43	1	83[73-93]%	53	0.2	93[85-100]%	43	1
**Enterococcus faecium daptomycin MIC median[IQR]**	2[2-3]	230	1	4[2-4]	215	<0.001	4[2-4]	91	<0.001
**Enterococcus faecalis daptomycin MIC median[IQR]**	1.25[1-1.5]	30	1	2[1-2]	51	0.043	1[1-2]	27	0.5
**Staphylococcus aureus daptomycin MIC median[IQR]**	0.38[0.25-0.62]	395	1	0.5[0.38-0.75]	409	<0.001	0.5[0.38-1]	250	<0.001
**CONS daptomycin MIC median[IQR]**	0.5[0.25-0.75]	43	1	0.5[0.5-1]	53	0.02	0.5[0.25-1]	43	0.7
**QC failure rate[CI]**	0.00[0.00-0.00]	0	1	0.01[0.00-0.01]	1	0.8	0.00[0.00-0.00]	0	1

[CI] = confidence intervals
CONS = Coagulase negative Staphylococci

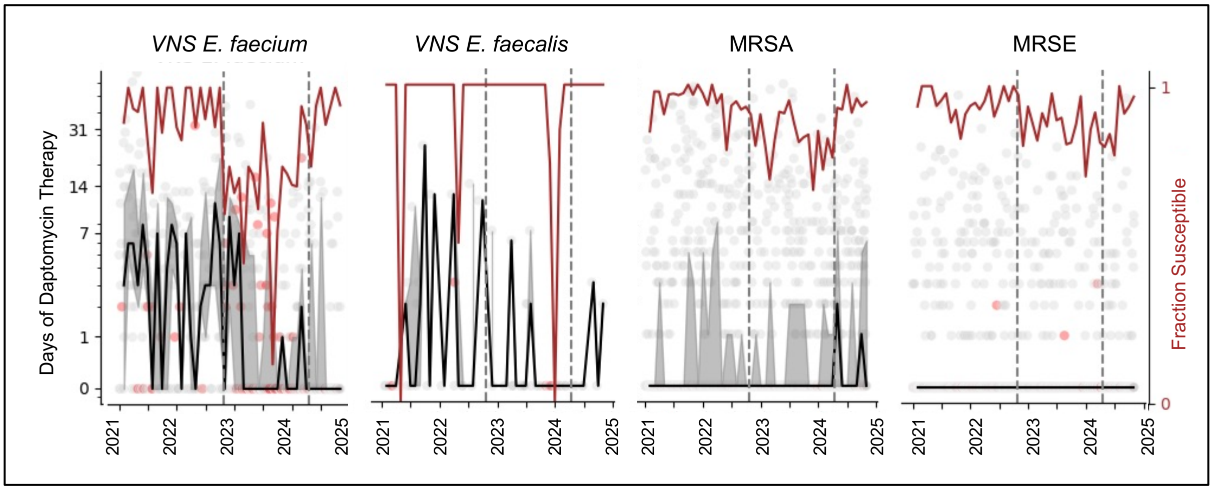

Supplemental Figure 1: Daptomycin prescribing data for BJH and SLCH patient encounters with cultures positive for resistant isolates of *Enterococcus faecium, Enterococcus faecalis , Staphylococcus aureus,* and *Staphylococcus epidermidis*. The number of antibiotic days for patient encounters at BJH and SLCH is demonstrated from 2021-2025. Dots indicate an index isolate categorized as S or SDD (gray) or not susceptible (red), and lines and shaded regions indicate the running median of days of daptomycin prescribed and interquartile ranges, respectively. For context, the red line demonstrates the fraction of daptomycin susceptible isolates and dashed grey vertical lines indicate the superimposed change point analysis. MR = methicillin resistant, VNS = Vancomycin nonsusceptible.
